# Mass Spectrometry Characterization of Honeydew Honey: A Critical Review

**DOI:** 10.3390/foods13142229

**Published:** 2024-07-16

**Authors:** Rosa Quirantes-Piné, Gavino Sanna, Andrea Mara, Isabel Borrás-Linares, Federica Mainente, Yolanda Picó, Gianni Zoccatelli, Jesús Lozano-Sánchez, Marco Ciulu

**Affiliations:** 1 Department of Analytical Chemistry, Faculty of Sciences, University of Granada, Avda Fuentenueva s/n, 18071 Granada, Spain; rquirantes@ugr.es; 2 Department of Chemical, Physical, Mathematical and Natural Sciences, University of Sassari, Via Vienna 2, 07100 Sassari, Italy; sanna@uniss.it (G.S.); amara@uniss.it (A.M.); 3 Department of Biotechnology, University of Verona, Strada le Grazie 15, Cà Vignal 1, 37134 Verona, Italy; federica.mainente@univr.it (F.M.); gianni.zoccatelli@univr.it (G.Z.); marco.ciulu@univr.it (M.C.); 4 Centro de Investigaciones Sobre Desertificaciòn, Ctra. Moncada-Naquera km 4.5, 46113 Moncada, Spain; yolanda.pico@uv.es; 5 Department of Food Science and Nutrition, Faculty of Pharmacy, University of Granada, Campus Universitario s/n, 18071 Granada, Spain; jesusls@ugr.es

**Keywords:** honeydew honey, mass spectrometry, volatiles, sugars, amino acids, proteins, phenolic compounds, elements, contaminants, toxins

## Abstract

Honeydew honey is produced by bees (*Apis mellifera*) foraging and collecting secretions produced by certain types of aphids on various parts of plants. In addition to exhibiting organoleptic characteristics that distinguish them from nectar honey, these honeys are known for their functional properties, such as strong antioxidant and anti-inflammatory activities. Despite their importance, they remain poorly characterized in comparison with flower honeys, as most studies on this subject are not only carried out on too few samples but also still focused on traditional chemical–physical parameters, such as specific rotation, major sugars, or melissopalynological information. Since mass spectrometry has consistently been a primary tool for the characterization and authentication of honeys, this review will focus on the application of these methods to the characterization of the minor fraction of honeydew honey. More specifically, this review will attempt to highlight what progress has been made so far in identifying markers of the authenticity of the botanical and/or geographical origin of honeydew honeys by mass spectrometry-based approaches. Furthermore, strategies devoted to the determination of contaminants and toxins in honeydew honeys will be addressed. Such analyses represent a valuable tool for establishing the level of food safety associated with these products. A critical analysis of the presented studies will identify their limitations and critical issues, thereby describing the current state of research on the topic.

## 1. Introduction

Honey is a sugar-based natural food with significant economic, dietary, and nutraceutical importance [1,2,3]. Bees (*Apis mellifera*) produce honey by foraging nectar or honeydew. Honeydew is the sugary substance that insects like *Metcalfa pruinosa* release on the bark or other parts of plants after assimilating the lymph. Although honeydew honey is less common and known than nectar honey, it is a very attractive beehive product due to its peculiar origin [4], organoleptic features, and functional properties, which make it an increasingly sought-after product.

Honeydew honeys result from a synergistic action between two insects. This leads to significant differences in their physicochemical, sensory, and functional properties [4]. The health-promoting properties of honeydew honey have been extensively reviewed elsewhere, with a focus on its antioxidant, antimicrobial, and anti-inflammatory effects [5]. The antioxidant capacity of honeydew honey has been assessed by different in vitro spectrophotometry assays, and, in most cases, it presented higher antioxidant potential than nectar honey, attributed to its richness in antioxidants such as phenolic compounds [6]. The antimicrobial effect of honeydew honey has also been demonstrated against Gram-negative bacteria, Gram-positive bacteria, and yeasts [6,7], also showing a more potent antibacterial activity than its nectar counterpart [8]. Regarding its anti-inflammatory properties, the effects of Bracatinga honeydew honey on inflammatory markers in macrophages have been reported and attributed to its phenolic content [9,10].

In addition, honeydew honeys are also useful for environmental biomonitoring because they are mainly produced in forest and woodland environments. This makes them a good source of information on the “health status” of these ecosystems [11,12]. Fir (sometimes identified as spruce or silver fir), pine, and oak honeydew honeys are the most common [13], but the production of hazelnut, eucalyptus, and citrus honeydew honeys has been established in the Mediterranean area [13]. A particularly well-known and investigated honey is the Bracantiga honeydew honey, typical of southern Brazil [14,15,16].

Despite its great interest, the production of honeydew honey is heavily influenced by human activity and the climate. The use of agrochemicals in crops limits the presence of honeydew-producing insects [17,18]. Additionally, global warming causes an overlap of nectar plant blooms, making it difficult to obtain pure honeydew honey [19]. Moreover, honeydew honey production is also hindered by growing competition with fake, adulterated, or poor-quality honey products. Indeed, honey counterfeiting is a current concern in the global context. These challenges can be met by implementing new strategies for the preservation and enhancement of honeydew honeys. One potential initial action could be the accurate declaration of the botanical origin on the label, as required by European regulations for geographical provenance [20]. Producers frequently label honeydew honey as “forest honeydew honey” or simply “honeydew honey” without specifying the precise botanical source. This is contingent upon the challenging nature of establishing the botanical source of honeydew honeys, in contrast to nectar honeys. It is clear that, in this context, the availability of precise and accurate analytical methods for the correct attribution of botanical and geographical origins is of fundamental importance for the valorization of beehive products still considered “minor”, such as honeydew honeys.

Many researchers have aimed to identify chemical markers and develop new analytical methods for honeydew honey’s origin attribution. Considerable effort has been invested in the identification of both botanical and geographical markers. However, it should be noted that it is necessary to consider both origins, as botanical markers may vary depending on the geographical origin. Among analytical methods, mass spectrometry (MS) has been one of the most employed in verifying the authenticity of honey [21]. Techniques such as liquid chromatography (LC) [22,23,24,25,26], gas chromatography (GC) [27,28,29], and inductively coupled plasma (ICP) [30,31,32] coupled with MS have made it possible to reliably determine both the floral [24,27,29,30,33] and geographical origins [31,33,34,35] of honeys. Additionally, they have enabled the precise determination of the molecular structure of health-promoting compounds [23,25,36,37] or, most frequently, contaminants [26,38,39,40,41,42,43,44], allowing for their accurate quantification.

To provide the reader with a comprehensive and up-to-date overview, this review will describe mass spectrometry-based methods for characterizing honeydew honeys. Specifically, this review will cover studies on sugars, amino acids and proteins, phenolic compounds, inorganic analytes, the volatile fraction, contaminants, and toxins.

## 2. Sugars

Honey is primarily composed of sugars. On average, sugars make up 80% of honey’s weight, with monosaccharides accounting for about 75% of the total composition of sugars [45]. The remaining amount consists of disaccharides (10–15%) and minor quantities of oligosaccharides. The composition of sugars depends mainly on the botanical and/or geographical origin of the honey, and secondly, it can be influenced by post-production phases. Glucose and fructose are the most abundant monosaccharides in honey, with the fructose/glucose ratio typically higher than 1 [46]. The only exceptions are honeys from countries with the abundant flowering of rape, ivy, or dandelion [47], whose nectar is richer in glucose than fructose. Moreover, honey contains various disaccharides, with sucrose being the most abundant [45]. Other disaccharides, such as trehalose, isomaltose, maltulose, turanose, and nigerose, may also be present, although in lower quantities than sucrose [48]. The concentration of trisaccharides in honey is often lower than that of disaccharides. Erlose, produced by honeybee invertase on sucrose, can reach a concentration of 6% in some cases. Melizitose, on the other hand, is virtually absent [49] (or present in very low amounts [50]) in blossom honeys but can exceed 20% in honeydew honeys [45]. For this reason, melizitose was initially proposed as a possible chemical marker for discriminating between honeydew and blossom honeys [48].

Differentiating between blossom and honeydew honeys based on sugar analysis can be quite challenging, especially if the quantification of melizitose is not considered. The glucose and fructose content in flower honeys is typically higher (70 ± 4%) than in honeydew honeys (58 ± 5%) [46,48]. However, the large uncertainty and variability in these values make discrimination based on this parameter difficult [51]. Although various studies suggest that the fructose/glucose ratio may be a reliable marker for distinguishing honeydew from blossom honeys [52], the analysis of a large sample of both types of honey revealed no statistically significant differences between the two types [46].

The presence and quantity of disaccharides do not permit the differentiation of honeydew honeys from flower honeys. However, certain trisaccharides, such as raffinose, have been proposed as potential differentiation markers [53]. Like melizetose, raffinose should be absent in blossom honeys but present in honeydew honeys. Unfortunately, additional studies confirm the lack of raffinose in several honeydew honeys [52,54]. Considering the state of the art, the application of MS-hyphenated methods to the trisaccharides contained in honeydew honey represents a pivotal tool for differentiating it from blossom honey and for identifying its botanical and/or geographical origin.

For instance, in a GC-MS study on Spanish honeys, melezitose and quercitol (sugar alcohol) were proposed as markers of *Quercus ilex* honeydew honey [55], whereas melezitose and erlose were indicated as markers of honeydew honeys of unspecified botanical origin [52]. In this context, it is worth mentioning the work of Terrab et al. [56], in which the combination of GC-MS analysis and chemometric techniques (PCA and SDA) allowed the correct classification of three honeydew honeys out of a total of ninety-eight samples of various botanical origins produced in northwestern Morocco. Notably, the level of classification accuracy for honeydew honeys was 100%, thereby confirming the reliability of the determination of minor sugars as a tool for discriminating honeydew honeys from nectar ones.

Blaško et al. developed an improved GC-MS method for the determination of melezitose in a sampling of Slovak and Austria floral and honeydew honeys [57]. The quantity of this analyte ranged from 10,600 to 26,100 mg kg^−1^ in honeydew honeys and from 96 to 2880 mg kg^−1^ in floral honeys, thereby confirming the suitability of melezitose as a reliable marker for the differentiation between honeydew honeys and floral honeys.

Beyond GC-MS approaches, HPLC-MS methods have also been used to distinguish honeydew honeys from unifloral and multifloral samples. In particular, high amounts of melezitose and raffinose were also found in honeydew honeys from Trentino Alto Adige, Italy, by using high-performance anion-exchange chromatography coupled with mass spectrometry (HPAEC-MS) [58].

As a concluding remark, while the identification of monosaccharides and disaccharides does not permit differentiation between honeydew and nectar honeys, this may be achieved by determining trisaccharides such as melezitose, erlose, or raffinose. Among the various trisaccharides, melezitose is undoubtedly the most reliable indicator, as it is present in honeydew honeys in concentrations that are several orders of magnitude higher than those observed in blossom honeys. In some cases, erlose and raffinose have also been used to achieve this discrimination. Nevertheless, the determination of sugars currently does not allow for the differentiation of the botanical origins of honeydew honeys or their geographic origin.

A selection of MS-hyphenated chromatographic methods devoted to distinguishing between honeydew honeys and blossom honeys according to their saccharide profiles is illustrated in Table 1.

## 3. Amino Acids and Proteins

Amino acids and proteins are among the minor components of honey since both classes account for less than 2% of the overall composition. In this regard, proline is the amino acid most abundant in honey, accounting for 50% to 85% of the total amount [59], while amylases, sucrase, α-glucosidase, and glucose oxidase are the most common enzymes [46]. Although not nutritionally important, both classes of compounds can provide useful information for product authentication. Thus, the amino acid profile is more suitable for the discrimination of the origin of honey than the protein composition. In particular, arginine, tryptophan, and cystine have been demonstrated to be present only in specific types of blossom honeys [60]. The quantity of both amino acids and proteins doubles in honeydew honeys with respect to blossom honeys; therefore, it can be used for their discrimination. Similarly, the amount of proline is also higher in honeydew honeys [5]. While these observations are typically insufficient for classification purposes, this result may be achieved through the determination of the free amino acids.

Senyuva et al. [60] demonstrated that the GC-MS-based determination of free amino acids, volatile compounds, saccharides, and water activity permitted the classification of seventy samples of Turkish honeys according to their botanical origin by PLS-DA. The study included eight floral sources (i.e., rhododendron, chestnut, thymus, eucalyptus, gossypium, citrus, sunflower, and multiflora) and honeydew honeys. Moreover, the determination of free amino acids alone has proven useful in certain instances for the classification of honeys according to their botanical or geographical origin.

In this context, an LC-MS-MS method without a prior derivatization step was employed to analyze the qualitative and quantitative profiles of 22 free amino acids in 65 floral honeys and 16 honeydew honeys from Eastern Europe and Central Asia [61]. The results demonstrated that, although it was not possible to identify a single amino acid as a specific floral marker, differences in the amount of certain analytes could be utilized for classification purposes. For instance, the content of phenylalanine was found to be higher in honeydew honeys from Poland than in the other samples under study. Furthermore, a cluster analysis (CA) enabled the differentiation of the geographical origins of honeydew honeys produced in two different regions of Poland.

The differentiation between honeydew and floral honeys based on their amino acid profiles has attracted the scientific interest of some researchers from Turkey. Silici and Karaman [62] distinguished between rhododendron honey and honeydew honey using an LC-MS method. Thirteen rhododendron samples from the Black Sea region and twelve honeydew samples from the Aegean region were analyzed, and 20 free amino acids were determined. High amounts of glycine and histidine are typical of honeydew honeys, whereas high concentrations of aspartic acid, cysteine, proline, and arginine identified rhododendron honeys. Also, Kivrak [63] attempted the quantification of 21 free amino acids in 51 unifloral honeys with 16 different floral origins and 7 samples of honeydew honeys produced in various Turkish regions. The samples were analyzed using ultra-performance liquid chromatography (UPLC)-ESI-MS-MS, and PCA allowed the samples to be differentiated according to their botanical origin.

The characterization of the amino acid profile of Bracatinga (*Mimosa scabrella* Bentham) honeydew honey has been a topic of recent research. Oliveira Costa’s research group first clarified, using a GC-MS approach, that the origin of some amino acids in Bracatinga honeydew honey is related to either *Apis mellifera* or plant-sucking insects [14]. Proline is exclusively related to the metabolic action of bees, while amino acids such as serine, asparagine, aspartic acid, and glutamic acid are related to plant-sucking insects’ metabolism. Additionally, the chemometric processing of the amino acid profile of Bracatinga honey was used to differentiate its geographic origin from different states of Brazil [16]. PCA made it possible to identify serine, asparagine, glutamic acid, and tryptophan as amino acids responsible for the geographic discrimination among samples from Santa Catarina and Paraná states. More recently, marker peptides of Bracatinga honey were identified by an untargeted LC-ESI-triple-TOF-MS-MS approach, followed by targeted quantification by LC-QqQ-MS-MS [64]. The study indicates that the peptide QNIDVVAR, one of the main royal jelly proteins, is the most useful for the discrimination between Bracatinga honeydew and floral honeys.

The use of proteomics for the identification of new authentication strategies for honeydew honeys was also proposed by Erban et al. [65]. In this study, conducted on 45 honey samples using label-free nano-LC-MS-MS proteomics, the presence of foreign amylases found in some samples revealed their adulteration. Several plant-related and, to a greater extent, honeybee-related proteins could be identified in that study. Moreover, a group of aphid-related proteins was identified as potentially eligible for the authentication of honeydew honeys. However, MS-based methods have not always been the optimal choice for honey authentication. As an example, the study of Brendel et al. [66] compared, for authentication purposes, the performance of medium-infrared (MIR) spectroscopy and the metabolomic profile obtained by Matrix-Assisted Laser Desorption Ionization (MALDI)-TOF-MS on multifloral and unifloral (i.e., canola, acacia, and honeydew) honeys. Among the classification models applied, PCA-LDA, PCA-k nearest neighbors (kNN), and soft independent modeling by class analogy (SIMCA) were utilized. The last model was found to be more effective than other class discrimination techniques in complex food authentication scenarios. Furthermore, the MIR approach demonstrated superior performance compared to the MALDI-TOF-MS method. The MIR correctly identified a higher percentage of samples of multifloral honey as outliers than MALDI-TOF-MS in the classification of the three unifloral honeys under investigation in this study.

A selection of MS-hyphenated chromatographic methods aimed to determine the amino acids or proteins in both nectar and honeydew honeys is illustrated in Table 2.

## 4. Phenolic Compounds

Phenolic compounds or polyphenols are secondary plant metabolites with several biological functionalities generally related to defense mechanisms against external threats. Therefore, these compounds are present in different parts of plants, and their derived products, such as nectar and sap, are later part of the minor composition of honey. Great attention has been paid to these phytochemicals due to their bioactive properties in humans, mainly antioxidant, anti-inflammatory, or anti-tumor effects. Since their presence and concentration are indicative of cultivar characteristics and ecosystem status, such as hydric stress, UV radiation, or pathogens, they could be proposed as potential biomarkers for product authentication, origin attribution, and/or classification.

Because of phenolic compounds’ polar nature, the most widely used analytical technique applied for their characterization in a wide range of samples along with honey is LC coupled with MS, normally following extraction procedures with polar solvents, including water. In honey samples, the most common phenolic compounds are phenolic acids and flavonoids, mainly from the sub-classes of flavones, flavanols, and benzoic and cinnamic acid derivatives [67,68,69]. In comparison to nectar honey, honeydew honey typically contains higher levels of phenolic acid derivatives but lower levels of flavonoids. Since honeydew honeys are often darker in color than nectar honeys, they exhibit higher antioxidant activity. However, there are some exceptions, as nectar honeys from oak, pine, chestnut, and heather have been documented to be darker in color than honeydew honeys, leading to higher antioxidant activities [4].

Focusing on honeydew honey samples, Seraglio et al. [70] developed a straightforward HPLC-ESI-MS-MS method for characterizing phenolic compounds in Bracatinga honeydew honey. Samples were processed by the dilute-and-shoot method without any additional clean-up or extraction steps. Nine samples from different regions of Brazil were diluted with water, vortex-mixed, shaken in an orbital shaker, centrifuged, filtered, and analyzed. To circumvent potential interference by polar species, the compounds eluted within the initial 1.9 min of the chromatographic run were discarded. The method was fully validated following Eurachem and European Commission guidelines. A total of 20 polyphenols were quantified, with 16 of them being present in all the analyzed samples. The most abundant compounds that were always identified were benzoic acid, 3,4-dihydroxybenzoic acid, and salicylic acid, followed by p-coumaric acid, ferulic acid, gallic acid, syringic acid, quercetin, and kaempferol.

The development of analytical methods for the assessment of the qualitative and quantitative profiles of phenolic species in honeydew honey has frequently been accompanied by an evaluation of their bioactivity. In this context, a qualitative and quantitative characterization of the phenolic fraction in fir honeydew honey was conducted by Matjan et al. [71]. Samples were first diluted with aqueous hydrochloric acid, and polyphenols were extracted using a C18 solid-phase extraction (SPE) cartridge. Of the 17 compounds identified, only 15 of them, classified as hydroxycinnamic acids, flavonols, flavones, and flavanones, were quantified. The study evaluated the bioactive potential of fir honeydew honey on TNF-α-induced MMP-9 expression and secretion from human keratinocytes. The results demonstrated that apigenin and kaempferol inhibit MMP-9 expression and production in a dose-dependent manner.

Kocyigit et al. [72] concentrated their efforts on the development and validation of an LC-MS method for the identification of phenolic species in 14 honey samples from Turkey. Before LC-MS analysis, the samples underwent liquid–liquid extraction (LLE) using curcumin as an internal standard. A total of 11 phenolic compounds were identified in honeydew honey, while only 2 were found in multifloral honey samples. The main compounds quantified in honeydew samples were salicylic acid, kaempferol, acacetin, caffeic acid, and apigenin. Among the samples from chestnut, pine, cedar, oak, and multiflora, only two were selected for bioactivity tests: Ida Mountains *Quercus pyrenaica* honeydew honey and Canakkale multifloral honey. These tests were conducted to assess the effects of honey on gastric adenocarcinoma cells, specifically in terms of DNA damage, apoptosis, and cell death. The bioactive anticancer action of the honeydew sample was found to be significantly higher than that of the multifloral honey, particularly at high doses, due to its higher phenolic content.

Moreover, phenolic compounds have been demonstrated to be a reliable tool for distinguishing between honeydew honeys and nectar honey samples, as well as for their differentiation according to their botanical origin. This distinction was frequently achieved through the support of supplementary analytical data and/or the application of chemometric approaches, which were employed to maximize the analytical information obtained. Trautvetter et al. [73] applied a targeted LC-MS analysis of ethyl acetate honey extracts to identify the presence of 33 phenolic compounds in 19 honey samples sourced from diverse botanical origins. These included four honeydew, four sunflower, three lime, five rape, and three clover honey samples. A total of 33 polyphenols were identified in the honeydew phenolic profile. Among these, the most significant were protocatechuic acid, abscisic acid, 4-hydroxybenzoic acid, β-phenyllactic acid, and chrysin.

Ciucure and Geană [74] developed an SPE-LC-MS method for the quantitative analysis of phenolic compounds in 28 blossom honeys and 5 honeydew honeys from Romania. The phenolic profile of honeydew honeys contains varying amounts of ferulic acid, syringic acid, p-coumaric acid, 3,4-dihydroxybenzoic acid, caffeic acid, pinocembrin, chrysin, galangin, quercetin, and apigenin. A PCA-HCA chemometric approach was employed to differentiate honeydew honeys from multifloral, acacia, and rape honeys. 3,4-Dihydroxybenzoic acid, syringic acid, trans-cinnamic acid, the total amounts of both phenolic and flavonoids, and the antioxidant capacity were found to be effective in distinguishing honeydew honey from all blossom honeys.

In the study conducted by Vazquez et al. [75], the phenolic fraction was quantified by a UAE-LC-MS-MS method. A total of 25 phenolic compounds were identified in 91 samples of honeys from Galicia (Northwest Spain). Honeydew, chestnut, eucalyptus, heather, blackberry, and multifloral honeys were analyzed. The most prevalent polyphenols identified in honeydew honeys were 3-hydroxyphenylacetic acid, gallic acid, protocatechuic acid, gentisic acid, and chrysin. The application of ANOVA and PCA to the results obtained by UAE-LC-MS-MS analysis in conjunction with the total phenolic content proved to be a valuable tool for the discrimination of botanical origin. The differentiation of honeydew honeys from floral honeys was accomplished based on the amounts of gallic acid, β-resorcylic acid, and protocatechuic acid.

However, the polyphenolic profile is not always a reliable indicator for differentiating honeydew honeys from other honeys of different botanical origins. This is exemplified by the study conducted by Nedic’ et al. [76], who evaluated the phenolic profile, antioxidant activity, electrical conductivity, melissopalynology profile, and antimicrobial activity for the classification of 27 honey samples (4 monofloral, 5 honeydew, and 18 polyfloral samples) from Serbia according to their botanical origin. The polyphenols were quantified using a UAE-SPE-LC-MS method, which enabled the identification of 6 phenolic acids, 13 flavonoids, and relevant glycosides. Among these, p-coumaric acid, caffeic acid, and pinocembrin were the most abundant polyphenols, yet no specific phenolic markers of botanical origin were identified. PCA was employed to differentiate the botanical origins of the honeydew samples based on physicochemical parameters, polyphenol contents, and antioxidant capacity. The PCA model revealed that the honeydew samples were grouped into one cluster, which was attributed to the melissopalynological fingerprint, the pH value, and the electrical conductivity.

On the other hand, the polyphenolic profile has also been successfully employed in the determination of the botanical and geographical origins of honeydew honeys in a study by Nešović et al. [77]. A previously developed UAE-SPE-LC-MS method was employed to determine the presence of 32 polyphenolic compounds in 20 floral honeys and 8 honeydew honeys sourced from three locations in northern Montenegro (Serbia). In total, 23 flavonoids, 9 phenolic acids, and their respective derivatives were quantified. The data indicate that the samples exhibit higher antioxidant activity than those from neighboring countries, including Serbia, Slovenia, Croatia, and Bulgaria. Furthermore, the quantities of luteolin, quercetin-3-O-galactoside, electrical conductivity, and turanose enabled the differentiation between honeydew and polyfloral honeys.

Spanish research groups have been active in the classification of honeys according to their botanical and geographical origins. In this context, García-Seval et al. [78] employed an SPE-LC-MS approach to assess the polyphenolic profile of a comprehensive array of floral and honeydew honeys sourced from Spain. A total of 110 floral honeys and 26 honeydew honeys from mountains, forests, and holm oak were analyzed. A satisfactory classification of the samples according to their botanical origin was achieved using PCA and PLS-DA. Furthermore, the cross-validation multiclass prediction values obtained for the differentiation of blossom and honeydew honey samples were excellent. Finally, the samples produced in the Mediterranean region were correctly distinguished from the others under study.

In their research, Hernanz et al. [79] focused their attention on oak honeydew honey. The polyphenolic profile of 58 Spanish samples of this honey was obtained using an SPE-LLE-LC-MS method. A total of 23 phenolic compounds were identified, including 7 benzoic acids, 4 hydroxycinnamic acids, and 12 flavonoids. Among these, syringic acid, naringenin, and galangin were the most abundant. The comparison of these data with those obtained from the same honey produced in other geographic localizations (e.g., Central Europe, Turkey, Greece, New Zealand, or Brazil) supports the contention that these three polyphenols could be considered as biomarkers for the authentication of Spanish oak honeydew honey.

The only contribution that represents a “voice out of the chorus” is that by Daher and Gülaçar [80], who employed SPME-GC-MS to identify polyphenols and other volatile analytes in 16 honey samples from diverse botanical and geographical origins. The use of polyacrylate fibers enabled the identification of 31 compounds, while 2,3-dihydrobenzofuran was employed as an external standard for quantification purposes. PCA enabled the differentiation between honeydew and nectar honeys. The higher concentration of salicylic acid in honeydew honeys compared to nectar honeys suggests that this could be a reliable tool for distinguishing between the two types of honey. Conversely, the absence of cinnamic acid appears to be a differentiating characteristic of honeydew honeys from the Pyrenees. However, given the limited number of samples considered in this research, further studies are necessary to verify these preliminary outcomes.

In addition, the phenolic profile of honeys has been employed as a tool to attempt classification according to the botanical origin of different honeydew honeys. This is exemplified by the contribution by Vasić et al. [81], who utilized different LC-MS approaches to discriminate the origin of 64 honeydew honey samples. In this research, 22 of the samples were derived from silver fir, 15 from evergreen oak, 4 from Hungarian oak, 6 from Montpellier maple, and 17 from conifers. A total of 52 phenolic compounds were identified through an SPE-UHPLC-LTQ OrbiTrapMS method, with 25 of these quantified through an SPE-UHPLC-DAD-MS-MS method. A pattern recognition analysis of the data from the phenolic compounds revealed that quercetin, naringenin, caffeoylquinic acid, hydroxyphenylacetic acid, apigenin, and genistein could be considered as potential markers of the botanical origin of honeydew honey. Conversely, a significant overlap between the five classes of honeydew honeys was evident from PCA.

As can be observed, LC-MS is the preferred technique for the analysis of phenolic compounds in honeydew honeys, just as LLE and SPE are the most popular extraction methods. While it is known that the phenolic profile of honeydew honeys, as well as nectar honeys, is closely related to their bioactive properties, its usefulness for authentication purposes has also been demonstrated. Among other components, salicylic acid, syringic acid, and some flavonoids seem to be the most promising for this purpose.

LC-MS-based studies related to the characterization of honeydew honeys described above are summarized in Table 3.

## 5. Minerals and Elements

Ash contains almost all the mineral components present in honey. The principal elements quantified in honey are K, P, Ca, Mg, and Na. However, other elements, such as Pb, Cd, Zn, Fe, Mn, Si, Al, B, Sn, Ba, Ag, Cr, Mo, and As, are also present in trace amounts [82]. The ash content of honey is influenced by its botanical and geographical origins, as well as by environmental factors such as the level of pollution at the production site [5]. In floral honeys, ash is typically ≤0.6%, which is well below the average amount of 1.2% normally measured in honeydew honeys [4]. While the total amount of ash is a recognized quality parameter of honeys [83], its elemental fingerprint is also widely used in honey authentication [84,85,86]. It reflects the pedological features of the areas in which the plants grow, as well as the different bioaccumulation pathways active for each element as a function of different botanical species involved in honey production [44]. Similarly, minor elements may be indicative of anthropogenic activities or pollution levels [44]. The chemometric management of large datasets from this approach allows for the differentiation of honey according to quality, food safety, or origin [32].

Basic analytical aspects concerning the determination and fractionation of elements in honey have already been treated in an exhaustive review published fifteen years ago [87]. Although atomic spectroscopy techniques such as flame atomic absorption spectroscopy (FAAS) or graphite furnace atomic absorption spectroscopy (GFAAS) were previously widely employed for the elemental characterization of honeys, contemporary methods rely on energy generation via inductively coupled plasma (ICP). In this context, hyphenated methods, such as atomic emission spectroscopy (ICP-AES) or, more effectively, mass spectrometry (ICP-MS), are the most commonly employed techniques [32]. Among these, ICP-MS is the most popular due to its high sensitivity, accuracy, rapidity, and productivity and its wide dynamic range. Before analysis, it is necessary to perform a suitable pretreatment of the sample, which involves the decomposition of the organic matrix of honey to minimize interference and increase sensitivity. This is frequently accomplished using a microwave-assisted oxidation performed with a nitric acid/hydrogen peroxide solution [88].

The classification of honeys from different origins according to their elemental content has gained attention from research groups worldwide. Among others, researchers from Brazil [89], Greece [90], Poland [30,91,92,93,94], Croatia [95,96], France [97], and Romania [97,98] differentiated between floral and honeydew honeys, considering both the botanical and geographical origins of floral and honeydew honeys.

In this regard, Polish honeydew honeys are among the most frequently studied. In their study, Madejczyk and Baralkiewicz [94] employed ICP-MS in conjunction with FAAS to ascertain the concentrations of 12 elements (Al, B, Ca, Cr, Cu, Fe, K, Mg, Mn, Na, Ni, and Zn) in both honeydew and rape honeys. In this study, only elements present in trace amounts (Al, B, Cr, Mn, and Ni) were analyzed by ICP-MS, while the remaining elements were determined by FAAS. CA of the data obtained revealed a correlation between the mineral composition and botanical origin. Honeydew honey exhibited higher concentrations of Mn, Al, Cu, K, Fe, and Ni than rape honey. Chudzinska and Baralkiewicz [30] further expanded this research to include buckwheat honey and analyzed additional toxic elements, such as Ba and Cd. Consequently, the concentration of 13 elements (Al, B, Ba, Ca, Cd, Cu, K, Mg, Mn, Na, Ni, Pb, Zn) was measured by ICP-MS in 55 honey samples derived from three distinct botanical sources (honeydew, buckwheat, and rape). CA allows for the observation of clear clusterization among honeydew and nectar honeys. Moreover, the same authors attempted to achieve classification according to both botanical and geographical origins using a larger sample (140 honeys from honeydew, buckwheat, and rape, gathered in 16 different zones of Poland) [91]. The authors used two chemometric methods: LDA and calibration and regression tree (C&RT). Both approaches permitted the accurate attribution of the botanical origin of each honey, with K and Mn identified as the most suitable descriptors. Conversely, no algorithm was able to classify both the geographical and botanical origins within the entire group of samples. Only LDA was able to achieve a satisfactory classification of the geographical origin of honeys using Mg, Al, and Mn as descriptors when samples with the same botanical origin were considered. It is therefore evident that, in this case, the weight of the botanical origin is greater than that of the geographical origin.

Drivelos et al. [90] employed a distinctive set of elements for classification based on ICP-MS measurements. In this case, rare-earth elements (REEs) were considered to achieve classification according to the botanical origin, geographical origin, and method of production (organic or traditional). In this case, 93 honey samples from diverse botanical origins (honeydew, buckwheat, and rape) and geographical locations (Poland, Greece, and other countries) were considered. CA was successfully employed to classify the samples according to their botanical origin, while DA was able to classify the samples according to both their botanical and geographical origins. Additionally, probabilistic neural network (PNN) and PLS models were able to accurately classify the samples according to their geographical origin. However, no algorithm was able to distinguish organic honey from conventional honey samples. Despite the comprehensive application of multivariate statistical techniques, the authors do not explicitly identify the specific elements responsible for each classification.

Silva et al. [89] determined the elemental profile of Brazilian Bracatinga honeydew honey for georeferencing purposes. The concentrations of 39 elements, including both major and trace elements, as well as REEs, were quantified using an ICP-MS method on 34 samples of Bracatinga honey from three different regions of Brazil. The concentrations of main and trace elements were more effective than those of REEs in grouping the samples. An LDA correctly classified 91.3% of the samples of Bracatinga honeydew honey according to their geographic origin. PCA identified Rb and Co as the main descriptors in such classification.

In addition, Magdas et al. [97] combined isotopic and elemental analyses for honey authentication based on geographical and botanical origins. The elemental fingerprints of 101 samples of unifloral honeys from Romania and France with 12 different botanical origins (common botanical sources: acacia, linden, honeydew, colza, and sunflower) were measured by an ICP-MS method. In addition to trace elements, toxic elements, and REEs, isotopic parameters were also measured, including δ^2^H and δ^18^O of the water extracted from honey, δ^13^C from raw honey, and δ^13^C and D/H ratios from the ethanol obtained through honey fermentation. LDA and SIMCA models were developed for the classification of honey based on geographical and botanical origins. Geographical classification was performed on the entire honey sample set. The LDA model permitted the effective classification of Romanian and French honeys with the aid of the D/H ratio, K, V, Cr, As, Nb, δ^2^H, Ce, and δ^13^C, which were identified as suitable classifying markers. The SIMCA classification yielded comparable outcomes. The percentage of differentiation reached 100% when the classification was performed within a single botanical origin. The classification according to botanical origin ranged from 94% (acacia, LDA) to over 80% (for acacia, honeydew, colza, and sunflower, SIMCA).

Oroian et al. [98] used a validated ICP-MS method to measure the fingerprints of 27 elements in 36 unifloral and honeydew honeys (acacia, sunflower, tilia, and honeydew) produced in northeastern Romania. Botanical classification was performed using PCA and DA. This chemometric approach allows the complete discrimination of the samples, with K, Mg, and Ca being the main descriptors for this differentiation.

The comparison between the total amounts of elements found in honeydew honeys and floral honeys has attracted the attention of research groups from Croatia. In their contribution, Vasic et al. [95], after an unsuccessful attempt to classify 64 samples of honeydew honeys with five botanical origins gathered in different zones in Croatia according to both their botanical and geographical origins, achieved a rough differentiation of the botanical origins of the samples based on their sugar profiles. PCA allows for the clear differentiation of Hungarian oak honeydew honeys from the remaining samples, using Mg, Mn, and Ba as descriptors. Additionally, a tentative differentiation can be made on PC1 between the conifer and silver fir honeydew honeys and the evergreen oak and Montpellier map honeydew honeys. This study did not establish any differentiators among the geographical origins of these honeys. Also, Bilandžić et al. [96] concentrated their efforts on the elemental fingerprints of honeys from Croatia. A total of 24 elements were quantified using an ICP-MS method in 28 honey samples from seven different botanical origins (multifloral, honeydew, and five unifloral) collected in southern Croatia. The authors did not provide a classification of the samples. However, the honeydew honey samples exhibited the highest concentrations of Al, As, Be, Cd, Co, Cu, K, Mn, Ni, Sb, Th, U, V, and Zn.

Other MS-based techniques have been used to authenticate the origins of both blossom and honeydew honeys. Bontempo et al. [99] analyzed stable isotope ratios (SIRs) using isotope ratio mass spectrometry (IRMS) and mineral element content in 265 blossom and honeydew honey samples collected throughout Italy. In this study, the comparison of carbon isotope ratios (δ^13^C) in combination with elemental composition was used to differentiate the botanical origin. Nevertheless, geographical differentiation was not possible due to limitations in the number of samples in each area. Finally, the use of an ICP-MS method in the determination of Pb and Cd present in honey samples of different botanical origins allowed Fraizzoli et al. [100] to make a valuable contribution regarding the sources of the combined analytical uncertainty. Reproducibility was identified as the main source contributing to the overall uncertainty of the method, which was applied to 13 selected samples of blossom and honeydew honeys produced in Italy. Although the concentrations of both elements are within the ranges typically found in Italian honeys and often below the amounts found in other countries, it is interesting to observe that honeydew honey samples present the highest amounts of Cd and Pb.

MS-based methods for the classification of honeydew honeys and blossom honeys by elemental fingerprinting are shown in Table 4.

## 6. Volatile Fraction

The volatile fraction defines the characteristic aroma of different types of honey. In principle, it may be a useful tool for identifying the botanical and/or geographic origins of honey [101,102]. For example, it can discriminate between honeydew and nectar honeys, as the aroma is generally stronger for the first [4]. The presence of many volatile compounds, which combine to create a unique scent for each type of honey, supports this idea.

Conversely, isolating and analyzing volatile compounds from complex matrices like honey can be a challenging task. Various approaches can be used, each with different degrees of selectivity and efficacy. The headspace solid-phase microextraction (HS-SPME) method is often preferred due to its simplicity, minimal handling, short extraction times, lack of need for organic solvents, and potential for quantifying numerous molecules [101]. On the other hand, the optimization of a large number of important parameters in the extraction of volatile compounds [103] from complex matrices could potentially affect both sensitivity and accuracy. This is because the qualitative and quantitative profiles of the volatile compounds obtained can vary significantly, even with minor changes in the extraction conditions. For these reasons, it is common to collect profiles obtained under different extraction conditions to gain a more comprehensive understanding of the volatile profiles of honeys [104]. Generally, the identification and quantification of extracted volatile species have been performed using a gas chromatography-coupled mass spectrometry (GC-MS) approach [104], but MS-based methods aimed at directly analyzing the headspace of honeys have also been proposed [105,106].

The literature contributions related to MS-based techniques for the characterization of volatile compounds in honeydew honeys can be divided into two categories: (i) the differentiation between honeydew and blossom honeys; (ii) the classification of honeydew honeys according to their botanical or geographical origins.

Castro-Vazquez and co-workers [107] conducted a qualitative and quantitative investigation using GC-MS on dichloromethane extracts of aqueous solutions of Spanish honeydew honeys from oak (two samples), holm oak (three samples), and forest (a mixture of oak and holm oak, four samples). They proposed trans-oak lactone, a volatile compound characteristic of oak wood, as a possible marker of the botanical origin of oak honeydew honey. The study also identified aminoacetophenone and propylanisole as characteristic markers of holm oak honeydew honey. Additionally, β-damascenone and phenylacetaldehyde were found to be the species mainly responsible for the characteristic aroma of forest, oak, and holm oak honeydew honeys. Caution should be exercised when generalizing the results described due to the limited number of samples analyzed.

Tananaki et al. [108] measured the volatile profile of pine honeydew honeys from Greece (22 samples) and Turkey (22 samples) using a purge-and-trap GC-MS method. They identified 77 compounds, with nonanale and octane being the most abundant ones. Fifteen compounds were found in both Greek and Turkish honeys, while nine species were found exclusively in Turkish honeys. Additionally, two volatile compounds were exclusively found in Greek honeys. Notably, 3-carene was present in all samples from Turkey but absent in all samples from Greece. Conversely, the exogenous species 1,4-dichlorobenzene was found exclusively in all samples from Greece and not in any samples from Turkey. As 1,4-dichlorobenzene is commonly used in Greece to fight wax moths, only 3-carene can be proposed as an intrinsic marker for the geographical origin of this honey. Kohonen self-organizing maps successfully differentiated the geographical origins of both pine honeydew honeys.

Bayraktar and Onoğur [109] analyzed twenty-four pine honeydew honey samples from three regions in Turkey (Marmaris, Datça, and Fethiye) using SPME-GC-MS. They identified fifty-one chemical species, with the most abundant being octanal, nonanal, decanal, dodecanal, pentadecane, nonadecane, nonanol, and 16-oxosalutaridine, overall accounting for 73% to 78% of the total volatile species. These species have consistently been found in honeys from all three regions. It should be noted that the candidate marker 3-carene, proposed by Tananaki et al. [108] for Turkish pine honeydew honey, was not identified in this study. Furthermore, 16-oxosalutaridine, which was proposed here as a candidate marker of authenticity for this honey, was not found in Tananaki’s study.

Geographical origin can also exert an effect on the volatile fraction of honeydews belonging to the same variety, and, in this context, it is worth mentioning the contribution of Karabagias et al. [110]. Thirty-four samples of *Quercus ilex* honeydew honey from seven regions of Greece were classified according to their geographical origin by HS-SPME-GC-MS target analysis, melissopalynology, and chemometric techniques. While eucalyptol, 1-decanol, and tetradecanoic acid ethyl ester are the volatile markers better describing Greek *Quercus ilex* honeydew honey, melissopalynological evidence was decisive in the attribution of the geographical origin of this honey. In a recent contribution [111], significant variations in the composition of specific markers (i.e., 2-butanone, 2-methylpropanal, ethyl acetate, and α-pinene) of the volatile fraction of honeydew honey were measured using an HS-SPME-GC-MS method during one-year storage under in-house conditions.

Lušić et al. [112] used an HS-SPME-GC-MS technique to establish the volatile profiles of three samples of fir honeydew honey and differentiate them from two other popular blossom honeys produced in Croatia: sage and lime honeys. The volatile fractions of the samples contained over 100 compounds, of which 45 were identified in fir honeydew honey. The chemical composition of Croatian fir honeydew honey differs from that of blossom honey due to the presence of acetonitrile, methyl-2-buten-1-ol, n-hexanol, 3-hexanol, 1-propyne, 2-furanmethanol, 5-methyl-2(5H)-furanone, 4-methylphenol, hexadecanoic acid, and methylheptanoate. These compounds are not found in sage and lime blossom honeys.

SPME-GC-MS allowed De la Fuente et al. [55] to discriminate one Spanish fir honeydew honey sample from twelve other blossom honeys based on the presence of terpenes like α-pinene, α-phellandrene, and eucalyptol. Soria et al. [113] used a purge-and-trap (P&T) method coupled with GC-MS to analyze the volatile fractions of twenty-two honeys from eight different botanical sources (eucalyptus, thyme, citrus, rosemary, heather, lavender, multiflower, and honeydew). The volatile fingerprint of the four honeydew honey samples is characterized by high amounts of aliphatic ketones and diketones (2-heptanone, 2,3-butanedione, 2,3-pentanedione) and alcohols (2-methyl-1-propanol, 2-methyl-1-butanol, 3-methyl-1-butanol). A stepwise discriminant analysis (DA) was used to differentiate the most represented botanical sources of honeys, namely, citrus, eucalyptus, and honeydew. The compounds dimethylsulfide, 2,3-butanedione, and dihydro-2-methyl-3(2H)-furanone were found to be effective in distinguishing honeydew honeys from citrus and eucalyptus honeys.

Senyuva’s research group [60] measured the profiles of volatile species and the concentrations of free amino acids and oligosaccharides in seventy samples of Turkish honeys from rhododendron, chestnut, honeydew, thyme, eucalyptus, cotton, citrus, sunflower, and multiflora. All six samples of honeydew honey were obtained from neighboring sites in southwestern Turkey. Their volatile fractions were analyzed using an HS-SPME-GC-MS approach. Among the more than 350 volatile species identified, nonanal and, in particular, α,α-dimethylphenylacetate could be proposed as markers of the botanical origin of honeydew honey from Turkey. Differentiation among the botanical origins of honeys was accomplished using partial least-squares (PLS) regression followed by linear discriminant analysis (LDA). While this study confirms that nonanal is an abundant volatile species present in Turkish honeydew honeys [108,109], the same is not true for α,α-dimethylphenylacetate, as it has never been identified in previous studies. In contrast, neither 3-carene [108] nor 16-oxosalutaridine [109], previously proposed as potential markers of the botanical origin of Turkish honeydew honey, were detected in this study.

Dymerski et al. [114] analyzed the volatile fingerprints of five samples each of unifloral blossom honeys, including acacia, linden, buckwheat, and rapeseed, as well as honeydew honey. Samples from various regions of Poland were analyzed using HS-SPME associated with bidimensional gas chromatography (GCxGC) and coupled with time-of-flight (TOF) MS. Out of the 329 compounds detected, 82 were identified by comparison with a real analytical standard. (E)-Nonen-2-al has been suggested as a potential marker for identifying the botanical origin of Polish honeydew honey. Additionally, a combination of four ethyl esters, 2,3-dimethylphenol, and 2,3-dimethylpyrazine can be used to classify the five samples based on their geographical origin.

Jánošková et al. used an HS-SPME-GCxGC-TOF-MS method [115] to analyze thirty-five Slovak honeydew honeys. Over 300 volatile compounds were detected, with approximately one-third of them identified and tentatively quantified. The volatile fraction of Slovak honeydew honey contains a high concentration of hydrocarbons, alcohols, aldehydes, ketones, terpenes, and benzene derivatives. Their concentrations are significantly higher than in the most common unifloral blossom honeys found in Slovakia. Additionally, 2-oxooctanoic acid, 4-oxapentanoic acid, allyl ester of acetic acid, and methyl ester of 2,6-dihydroxybenzoic acid, which are always present in honeydew honey, are never identified in blossom honeys. Therefore, the authors suggested them as potential markers of origin for Slovakian honeydew honeys.

A method of differentiation between the botanical origins of two honeydew honeys from Greece was proposed by Karabagias et al. [116]. An HS-SPME-GC-MS method applied to a large sample (119 unifloral honeys collected from fourteen different geographical regions) allowed the discrimination of fir honeydew honeys (31 samples) from pine honeydew honeys (39 samples) and three other Greek unifloral blossom honeys based on their larger amounts of C6-C14 ethyl esters (hexanoic, heptanoic, octanoic, nonanoic, decanoic, dodecanoic, tetradecanoic derivatives). The exogenous species 1,4-dichlorobenzene, suggested by Takanaki [108] as a potential marker of Greek honeys, was never found in this sampling.

No method for the geographical differentiation of honeys with the same botanical origin has been proposed. Siegmund et al. [117] analyzed the volatile compounds in eight honey samples, each with a different botanical origin (dandelion, fir tree, linden tree, chestnut tree, robinia, orange, lavender, and rape) from Austria and Croatia. They used one- and two-dimensional HS-SPME-GC-MS techniques. The biplot of the principal component analysis (PCA) differentiated the cluster of the honeydew honeys (i.e., fir, chestnut, and linden honeys) from the blossom honeys. Among the 76 volatile species identified, the group of honeydew honeys was correlated with the significant presence of butanoic acid (mainly present in fir honeydew honey), diacetyl, and 2-aminoacetophenone (both strongly correlated with chestnut honeydew honey). Also, in this case, it should be noted that the reliability of these results could be strongly affected by the small number of samples analyzed for each type of honey.

Yang et al. [118] analyzed more than 250 honeys from Corsica using both HS-SPME-GC-FID (Flame Ionization Detector, FID) and HS-SPME-GC-MS techniques. The chemometric elaboration allowed the differentiation between honeydew honey and blossom honey based on the richness of 3-furaldehyde in the category of “honeydew maquis”. In addition, the volatile profile of *Metcalfa* honeydew honey was reported for the first time.

Karabagias et al. [119] used an HS-SPME-GC-MS approach to identify and semi-quantify 72 volatile compounds in 32 samples of Greek honey, including 6 honeydew honeys from Arkadia. The analysis revealed only small amounts of C8-C10 linear aldehydes, ethyl esters, and alkanes, furfural, thymol, and α-pinene in these honeys, with none of these species being unique to this honey.

Although analyses were performed mainly using the SPME-GC-MS technique, the observed differences in the optimization of the extraction phase likely reflect differences in both qualitative and quantitative responses obtained from analyzing honeys, even from the same origin [60,108,109,116].

More recently, Quintanilla-López et al. [120] aimed to classify Spanish honeys from five botanical origins based on their volatile fingerprints using an HS-SPME extraction method followed by a Direct Injection Mass Spectrometry (DIMS) technique. The chemometric treatment of the HS-SPME-DIMS data using a partial least-square (PLS)–discriminant analysis (DA) successfully classified 22 honey samples from acacia, citrus, eucalyptus, honeydew, and rosemary. The study identified 35 volatile compounds. The most represented classes in honeydew honey were alcohols, aldehydes, and ketones. The specific volatile fingerprint of honeydew honey was characterized by high amounts of 2-phenylethanol, isophorone, 2-methyl-1-butanol, and 3-methyl-1-butanol and low amounts of furfural when compared to other blossom honeys. Dimethylacetophenone, which is abundant in honeydew honey, was not found in any of the blossom honeys studied. Therefore, it could be suggested as a potential marker for identifying the botanical origin.

In certain cases, botanical discrimination has been accomplished without extracting volatile species from the headspace. For instance, Langford et al. [105] conducted an analytical study to distinguish honeydew from nectar honeys. Unlike the usual HS-SPME-GC-MS method, they directly analyzed the headspace of the sample using selected ion flow tube-mass spectrometry (SIFT-MS). This emerging technique was applied to eight different blossom unifloral honeys and the beech honeydew honey from New Zealand. The initial findings, based on the analysis of one sample per botanical origin, suggest the potential use of the SIFT-MS technique in combination with multivariate statistical analysis to distinguish the renowned unifloral manuka honey from its most prevalent contaminant, such as beech honeydew honey.

Also, in the study conducted by Schufried et al. [106], the headspace of seventy honey samples from seven botanical origins (i.e., citrus, chestnut, sunflower, honeydew, robinia, rhododendron, linden tree) was directly analyzed using proton-transfer-reaction time-of-flight-mass spectrometry (PTR-TOF-MS) technique. Although there were minimal differences among the volatile profiles of the samples measured, the adoption of chemometric techniques such as stepwise LDA and a probabilistic neural network (PNN) allowed for their correct classification according to their botanical origin.

More recently, Manousi et al. [121] compared the performance of a traditional solid-phase microextraction (SPME) method with that of an SPME method using an arrow fiber combined with two-dimensional gas chromatography–mass spectrometry (GC×GC–MS). The results showed that the arrow-fiber-based SPME-GC×GC–MS method was more sensitive, more precise, and able to detect a greater number of volatile compounds than the traditional SPME-based method. The study successfully differentiated between pine honeydew honey from Greece, forest honeydew honey, and floral honey, all purchased in Austria. The forest honeydew honey was characterized by high amounts of octanoic acid, 3,5,5-trimethylcyclohex-2-enone, and α-methyl-α-[4-methyl-3-pentenyl]-oxiranemethanol, while nonanal and nonanoic acid were identified as possible markers for pine honeydew honey. The significance of the attributions made is greatly reduced due to the paucity of samples analyzed and the absence of definite information about their places of origin. Anyway, it is worth noting that nonanal and nonanoic acids are among the most abundant volatile compounds identified in pine honeydew honey from Greece.

Some literature contributions focus on determining the volatile fingerprints of honeydew honeys from specific botanical origins. Jerkovic’s research group has conducted research in this area [122,123,124]. They initially characterized the volatile fraction of oak honeydew honey [122] using GC-MS methods coupled with HS-SPME or ultrasonic-assisted extraction (UAE) with either dichloromethane or pentane/diethyl ether as the extracting solvent. The HS-SPME extracts contained mostly terpenes, specifically cis- and trans-isomers of linalool oxides. Meanwhile, the UAE extracts were primarily composed of derivatives from the shikimic pathway, with phenylacetic acid being the most abundant species. Additionally, high amounts of 1-(2-furyl)-2-hydroxyethanone, benzoic acid, methyl syringate, 4-methyl-2,6-bis(1,1-dimethylethyl)phenol, 4-hydroxybenzoic acid, and tricosane were found.

The group later utilized a UAE-GC-MS approach to identify the volatile fingerprints of Salix [123] and fir [124] samples. It was discovered that all of these honeydew honeys contained high levels of phenylacetic acid, hexadecan-1-ol, 4-hydroxyphenylacetic, and 4-hydroxycinnamic acid, as well as palmitic acid. The analysis revealed that Salix honeydew honey also contains significant amounts of oleic acid and octadecane-1-ol. On the other hand, the volatile fraction of fir honeydew honey is mainly composed of phenylacetic acid, 4-hydroxyphenylacetic acid, 4-hydroxycinnamic acid, hexadecane-1-ol, and palmitic acid.

Kus et al. [125] conducted a comprehensive analysis of Polish fir honeydew honeys. The analysis included the characterization of its chemical–physical parameters, sensory attributes, and volatile and polyphenolic fractions. The study aimed to assess the honey’s chemical profile and identify potential markers of botanical origin. The authors performed both UAE-GC-MS and HS-SPME-GC-MS analyses of the volatile profiles of five samples. The analysis of UAE-GC-MS extracts revealed that the most abundant compounds were higher aliphatic hydrocarbons and alcohols, as well as phenylpropanoids. Conversely, HS-SPME-GC-MS analysis showed that the most abundant species were benzaldehyde, phenylacetaldehyde, and linalool derivatives. It is worth noting that none of the main volatile species found in fir honeydew honeys from other countries [55,112,116] were detected in this study. Anyway, since significant amounts of 3,4-dihydroxybenzoic acid were detected in the Polish samples, the authors suggested it as a potential marker for identifying the botanical origin of fir honeydew honey. An HPLC-DAD method was used to measure it instead of a GC-MS approach.

The poor reproducibility of the composition of the main volatile species observed in the analysis of honeydew honeys, even when they come from a common botanical and/or geographical origin, raises concerns about the general applicability of this approach. The major reasons for the irreproducibility of literature studies are inadequate sampling, insufficient verified information on the botanical origin, and significant variations in the efficiency and selectivity of extraction methods. In conclusion, discrimination based on the volatile fingerprints of honeydew honeys and blossom honeys produced in the same area is currently the only reliable outcome. Other results are only applicable under specific conditions reported in each research.

A selection of GC-MS-based studies regarding the characterization of honeydew honeys is illustrated in Table 5.

## 7. Contaminants and Toxins

In the previous sections, the authenticity of honeydew honeys has been established by determining different classes of compounds using MS-based analytical methods. Except for toxic elements or heavy metals, the food safety of honeydew honey has not been discussed. Other compounds, such as insecticides, pesticides, antibiotics, toxins, and their relevant metabolites, could, in principle, threaten the safety of honeydew honeys. The final section of this review will address this issue and the role of MS-based methods in the accurate and sensitive determination of these hazardous species. To accomplish this aim, LC-MS and GC-MS methods were mainly developed and tested on real samples of honeydew and/or honeydew honeys.

The main contaminants that could be present in the honeydew composition are those coming from systemic insecticides widely used to manage insect pests in agriculture. Quesada et al. [126] used LC-MS-MS to assess the effect of the application of imidacloprid and spirotetramat to soil or foliage on the excretion of these insecticides by striped pine scales through honeydew. Their analyses concluded the presence of both insecticides in a non-metabolized form in honeydew after four days of both soil and foliar treatments. In addition, striped pine scales excrete insecticides in honeydew even when the toxicant severely reduces honeydew production. Thus, honeydew excretion is a mechanism of bioaccumulation and has the potential to harm honeydew-feeding organisms like bees.

The translocation of two other insecticides commonly used in Integrated Pest Management programs, namely, pymetrozine and flonicamid, was also studied by Calvo-Agudo et al. [127] using LC-MS-MS measurements. These insecticides can also be present in honeydew excreted by hemipterans feeding on treated *Planococcus citri* trees. The presence of insecticides in hemipteran honeydew depends on their species. The results of this study suggest that honeydew-producing species that are tolerant or resistant to insecticides may excrete contaminated honeydew for longer periods. Therefore, the environmental risk to beneficial insects and consumers of honeydew-based products is higher than that of contaminated nectar and should be considered in future environmental risk assessments.

The consequences of insecticide treatment for pest control on honeydew honey safety were investigated and reported by Underwood et al. [128]. The insecticide dinotefuran has been used in the United States to control the spotted lanternfly, a plant-feeding insect that colonizes and severely damages trees such as the Tree of Heaven. The spotted lanternfly produces abundant honeydew, which attracts beneficial insects such as honeybees. For this reason, the study was designed to assess the risk of dinotefuran translocation to bees and beekeeping products. Therefore, honeybee colonies in areas with high densities of dinotefuran-treated trap trees were identified, and samples of honeydew, honeydew honey, bees, and beeswax were collected, extracted by the Quick, Easy, Cheap, Effective, Rugged, and Safe (QuEChERS) method, and analyzed by an LC-MS-MS method to quantify the target analyte. Although very low levels of dinotefuran were found in honeydew samples, the concentration of this insecticide was below the detection limit in the other matrices.

Furthermore, Brugnerotto et al. [43] used a GC-MS method to measure the concentrations of seven pesticides (atrazine, chlorpyrifos, α-endosulfan, τ-fluvalinate, chlorfenvinphos, chlorfenvinphos, bromopropylate, and coumaphos) in 28 samples of Bracatinga honeydew honey produced in two Brazilian states. Traces of τ-fluvalinate were found in only one honey sample, while low levels of atrazine were found in some samples produced in the state of Santa Catarina. However, the concentrations found in those samples were less than 20% of the maximum residue limits established for honey.

On the other hand, the most important toxin potentially present in honeydew honey is tutin, an oxygenated sesquiterpene picrotoxane neurotoxin that may be found in some honeydew honeys produced in New Zealand. Tutin can enter honey when bees collect honeydew shed by an insect (*Scolypopa australis*) that feeds on the sap of tutu, a poisonous shrub that contains it. Tutin and other related compounds, mainly glycoside derivatives, were detected and quantified by Larsen et al. [129] in three poisoned or non-compliant honey samples using an LC-MS-MS method, while NMR spectrometry was used to characterize the tutin-derived structures. Furthermore, Watkins et al. [130] also used an LC-MS-MS method to determine tutin, its hydroxylated derivative hyenanchin, and the monoglucoside and diglucoside forms of tutin in a toxic New Zealand honey. In this study, samples of different parts of tutu, *Scolypopa australis*, and its honeydew were also analyzed, and the results confirmed that all of these picrotoxanes, including tutin derivatives, were of plant origin and not metabolites produced by the insect.

As a summary, Table 6 reports the selected MS-based studies on the determination of contaminants and toxins in honeydew honeys.

## 8. Data Treatment

As explained above, one of the biggest issues concerning studies aimed at characterizing honeydew honeys, not only by mass spectrometry but also by other techniques, is the small number of samples on which such studies have been conducted. Alongside this, however, there are also examples in the scientific literature of MS-based studies conducted on a large number of honeys. In this context, the use of the correct statistical and/or chemometric approach has proved essential in order to obtain useful information from extensive and difficult-to-interpret datasets derived from techniques such as GC-MS, LC-MS, LC-MS/MS, etc. In these cases, the overall analytical information is often multidimensional in nature, as it is given by peak areas, retention times, and *m*/*z* ratios.

Principal component analysis (PCA) represents, in this sense, the first approach to be adopted to verify the presence of outliers and to visualize, in the first instance, whether the discrimination of the samples on the basis of the selected classes is possible. This type of approach proved useful both in the discrimination between honeydew honeys and other honeys [56,64,75,76,78,80,95,98,120] and, in a few cases, in the georeferencing of the samples [19,89].

In addition to a mere visualization aimed at verifying the possible presence of clusters associated with the botanical and geographical origins under study, the application of classification algorithms is fundamental in this type of research in order to check whether the information obtained through MS-based techniques can actually be used for practical purposes. Among all methods used, linear discriminant analysis (LDA) is the one that finds the greatest application in the classification of honeydew honeys. This type of approach was effective in the classification of geographical origin [89,90,91,97], a particularly difficult task to perform, especially if nearby regions are taken into consideration. In addition to LDA-based approaches, soft independent modeling of class analogies (SIMCA) proved to be useful for classification purposes [66,97].

Moreover, good classification results for honeydew honeys were obtained by the combination of partial least-squares regression and discriminant analysis (PLS-DA), which is normally used when the predicted variable is categorical. It is noteworthy to mention the great power of this chemometric approach in the classification of botanical origin based on untargeted fingerprinting of the samples. Although these kinds of studies are still scarce in the case of honeydew honeys, the aforementioned study by Garcia-Seval [78] offers a good example of how PLS-DA can offer considerable help in classifying honeys on the basis of their floral origin and area of provenance.

## 9. Conclusions

This review presents the results of studies employing mass spectrometry methods to characterize both the organic and inorganic minor components of honeydew honeys. The presented findings indicate a significant lack of knowledge regarding the composition of various honeydew honeys and their role in classification issues. Moreover, the analytical methods employed frequently lack sufficient validation. As expected, the literature indicates that chromatographic methods coupled with MS are the primary techniques employed to determine the organic component. Moreover, ICP-MS methods have been employed in nearly all studies devoted to the characterization of inorganic trace elements. Generally, the classification of samples according to their botanical or geographical origin is a challenging task. The limited number of studies on the determination of the minor saccharide component, amino acids, and proteins in honeydew honey have demonstrated that these categories of analytes contribute very little to the resolution of classification problems, except when they are used in conjunction with other analytical approaches. The only significant exception is the trisaccharide melezitose, which has been identified as a reliable marker for differentiating honeydew honey from nectar honey. The LC-MS determination of polyphenolic compounds and the ICP-MS determination of the elemental fingerprint of honeydew honey yielded data that were not comparable with different studies performed on the same matrix. This is due to both disparate analytical approaches and, mainly, the paucity of samples analyzed, which precludes the drawing of general conclusions. Consequently, the classification of samples according to their botanical origin has proven to be a significant challenge, while classification according to their geographical origin is even harder. A comparable situation, although even more pronounced than the previous one, can be observed in the determination of the volatile fraction, frequently conducted by GC-MS methods with HS-SPME preconcentration. In this case, classification according to geographical origin is often perceived as an “impossible task”. On the other hand, the actual level of food safety for honeydew honey is satisfactory, as the levels of both inorganic and organic toxicants are below the highest permitted levels. Nevertheless, certain specific circumstances, which are essentially attributable to the potential bioaccumulation of insecticides, pesticides, and toxins, will require close monitoring in the near future.

## Figures and Tables

**Table 1:** Selected MS-hyphenated chromatographic methods for the determination of saccharides in honeydew honeys.

Sample Pretreatment	Chromatographic Conditions ^a^	Samples (Country of Origin)	Main Outcomes	Reference
Oximation and trimethylsilylation derivatization procedure	GC-MS, SPB-1, 30 × 0.25, 0.25	1 fir honeydew, 1 *Quercus ilex* honeydew, and 11 blossom honeys (Spain)	Fifteen disaccharides and seven trisaccharides were identified in the honeys under investigation. Melezitose and the polyalcohol quercitol were proposed as markers for *Quercus ilex* honeydew honeys from Spain.	[55]
Oximation and trimethylsilylation derivatization procedure	GC-MS, OV-1, 25 × 0.25, 0.25	98 honey samples, 3 honeydew honeys (Morocco)	Two monosaccharides, eight disaccharides, and three trisaccharides were quantified. The application of stepwise discriminant analysis (SDA) permitted the correct attribution of 100% of the honeydew honeys.	[56]
Derivatization procedure with hexamethyldisilazane and N, O-bis(trimethylsilyl) trifluoroacetamide	GC-MS, DB-5MS, 5 × 0.25, 0.25	11 blossom and 7 honeydew honeys (Slovakia and Austria)	The quantitative determination of melezitose in honey samples provides evidence that it can be used as a reliable marker to distinguish honeydew honeys from blossom honeys.	[57]
None	HPAEC-MS, CarboPack PA-10, 0.25 × 2, 10 Mobile phase: NaOH gradient Ionization mode: negative	23 multifloral, 4 acacia, 4 dandelion, 8 rhododendron, and 4 honeydew honeys (Trentino Alto-Adige, Italy)	High amounts of melezitose and raffinose were also found in honeydew honeys.	[58]

^a^ Column model, length, m × internal diameter, mm; film thickness, µm.

**Table 2:** Selected MS-hyphenated methods for the determination of amino acids or proteins in honeydew honeys.

Sample Pretreatment	Chromatographic Conditions ^a^	Samples (Country of Origin)	Main Outcomes	Reference
Derivatization was made according to the Z:faast GC-MS kit for free amino acid analysis.	GC-MS, column was provided by the EZ:faast GC-MS kit for free amino acid analysis.	21 Bracatinga honeydew honeys (Brazil)	Proline is exclusively related to the metabolic action of bees, while amino acids such as serine, asparagine, aspartic acid, and glutamic acid are generally related to the metabolism of plant-sucking insects.	[14]
Derivatization was performed according to the Z:faast GC-MS kit for free amino acid analysis.	GC-MS, column was provided by the EZ:faast GC-MS kit for free amino acid analysis.	28 Bracatinga honeydew honeys (Brazil)	Chemometric processing of the amino acid profile of Bracatinga honey was used to differentiate its geographical origin from different states of Brazil.	[16]
Acidification with 0.2 mM acetic acid solution.	LC-MS, Zorbax RP 0.1 × 2.1, 3.5. Isocratic elution. Mobile phase: 0.01 mM HAc + 0.2% formic acid. Ionization mode: positive.	70 blossom and honeydew honeys (Turkey)	Although the amino acid profiles of honeys are unable to distinguish their origins, a combined dataset of amino acids, volatiles, saccharides, and water activity measurements allows the floral origin of Turkish honey to be accurately predicted.	[60]
Extraction using 0.1% formic acid in the water/methanol mixture (8:2, *v*/*v*), clean-up with C18 or GCB sorbents.	LC-MS-MS, KINETEX HILIC (i) 0.05 × 2.1, 1.7 μm; (ii) KINETEX RP-C18, 0.05 × 2.1, 2.6; (iii) Hypercarb, 0.1 × 2.1, 5.0. Gradient elution. Mobile phase: phase A: water + 0.2% formic acid + 20 mM ammonium formate; phase B: acetonitrile. Ionization mode: positive.	65 blossom and 16 honeydew honeys (Eastern Europe and Central Asia)	The concentration of specific amino acids may be exploited for the purpose of classification. A cluster analysis enabled the differentiation of the geographical origin of honeydew honeys produced in two different regions of Poland.	[61]
Acidification with 0.2 mM acetic acid solution.	LC-MS, Zorbax RP, Narrow-Bore 100 × 2.1, 3.5. Isocratic elution. Mobile phase: 0.01 mM acetic acid. Ionization mode: positive/negative.	13 rhododendron, 12 honeydew honeys (Turkey)	PCA and HCA were used to characterize and classify honey samples. The most abundant amino acids in rhododendron honeys were aspartic acid, lysine, and arginine, while those in honeydew honeys were, after proline, lysine, arginine, and histidine.	[62]
Sample dilution with 20% methanol solution (*v*/*v*) acidified with 0.1% formic acid (*v*/*v*).	UPLC–ESI–MS-MS, UPLC BEH C18, 0.1 × 2.1, 1.7. Gradient elution. Mobile phase: 0.5% aqueous formic acid (A); methanol/water (50:50, *v*/*v*) containing 0.5% formic acid (B). Ionization mode: positive	51 unifloral honeys (16 different floral origins) and 7 honeydew honeys (Turkey)	Twenty-one amino acids were quantified in this sampling. PCA allowed the samples to be differentiated according to their botanical origin.	[63]
Honey samples were mixed with extraction buffer (100 mM ammonium bicarbonate, 5 mM dithiothreitol, and 4 M urea, pH 8.2); hence, the proteins were extracted and concentrated.	LC-ESI-Triple-TOF-MS-MS, Gradient elution. Mobile phase: C18 column, 0.05 × 0.5, 2.7 eluent A (0.1% formic acid); eluent B (acetonitrile with 0.1% formic acid). Ionization mode: positive	12 honeydew honeys and 12 blossom honeys (Brazil and Germany)	The QNIDVVAR peptide is useful for discriminating between Bracatinga honeydew and floral honeys.	[64]
Samples were dissolved in ultrapure water, cleaned using PD MidiTrap G-25 columns, and concentrated by lyophilization.	Label-free nanoLC-MS-MS, EASYSpray PepMap C18, 0.50 × 75, 2. Gradient elution. Mobile phase: (A) water and 0.1% formic acid. (B) CH_3_CN and 0.1% formic acid.	45 honey samples (Czechia and other countries)	This approach is useful for the accurate identification of amylases/diastases added to mask reduced enzyme activity. In addition, it has been shown that aphid-related proteins, such as those of honeydew-producing insects, can be identified in honey and may be suitable for the authentication of honeydew honeys.	[65]
Samples were dissolved in ultrapure water. Then, equal volumes of honey samples (10 wt%) and matrix (4-chloro-α-cyanocinnamic acid, 5 mg/mL, dissolved in 90% aqueous acetonitrile containing 0.1% TFA) were mixed.	MALDI-TOF-MS MIR spectroscopy. Ionization mode: positive.	69 unifloral honeys from acacia and canola, and honeydew honeys (Europe)	A comparison of MIR spectra and metabolomic profiles obtained by MALDI-TOF-MS for authentication purposes showed the superior performance of MIR in terms of classification accuracy. The SIMCA model provides the best performance with respect to PCA-LDA and PCA-kNN approaches.	[66]

^a^ Column model, length, m × internal diameter, mm; film thickness, µm.

**Table 3:** Selected MS-based studies for the investigation of phenolic fraction in honeydew honeys.

Extraction Technique	Chromatographic Conditions ^a^	Samples (Country of Origin)	Main Outcomes	Reference
Dilute and shoot	LC-MS. VENUSIL C18 (0.1 × 2.1; 3). Gradient elution; mobile phases: (A) water with 0.1% formic acid, (B) acetonitrile with 0.1% formic acid. Ionization mode: positive/negative.	9 Bracatinga honeydew honeys (Brazil)	Twenty phenolic compounds were detected and quantified, the most abundant of them being benzoic acid, 3,4-dihydroxybenzoic acid and salicylic acid.	[70]
SPE	LC-MS. Pursuit XRs C18 (250 × 9.4, 5). Gradient elution; mobile phases: (A) water with 1% formic acid, (B) acetonitrile. Ionization mode: negative.	1 fir honeydew honey (unspecified geographical origin)	Seventeen phenolic compounds classified as hydroxycinnamic acids, flavonols, flavones, and flavanones were detected, and fifteen were quantified.	[71]
LLE	LC-MS-MS. Fortis C18 (0.15 × 3.0, 5). Temperature: 30 °C. Gradient elution; mobile phases: (A) water with 0.1% formic acid, (B) acetonitrile with 0.1% formic acid. Ionization mode: negative.	14 honey samples: chestnut, pine, cedar, oak, multifloral. For detailed analysis, 2 varieties selected: Ida Mountains *Quercus pyrenaica* honeydew honey and Canakkale multifloral honey (Turkey)	Eleven phenolic compounds were detected in honeydew honey, the major compounds quantified being salicylic acid, kaempferol, acacetin, caffeic acid, and apigenin.	[72]
LLE	UPLC-MS. Acquity UPLCTM BEH C18 (0.1 × 2.1, 7 μm). Gradient elution; mobile phases: (A) 2% acetic acid, (B) methanol. Ionization mode: negative.	4 honeydew, 4 sunflower, 3 lime, 5 rape, and 3 clover honeys (unspecified geographical origin)	Honeydew samples contain 33 polyphenols, with highlighted ones being protocatechuic and abscisic acids, 4-hydroxybenzoic acid, β-phenyllactic acid, and chrysin.	[73]
SLE	UHPLC-DAD-ESI-MS. Accuacore PFP (0.05 × 2.1, 2.6, and 0.10 × 2.1, 2.6). Gradient elution; mobile phases: (A) water with 0.1% formic acid, (B) methanol with 0.1% formic acid. Ionization mode: negative.	8 acacia, 10 oilseed rape, 5 multifloral, and 5 honeydew honeys (Romania)	Thirty-one phenolic compounds were qualitatively and twenty-four quantitatively determined in honey samples, with the contents of 3,4-dihydroxybenzoic, syringic, and trans-cinnamic acids being representative of honeydew honey.	[74]
Vortex	LC-MS-MS. Kinetex C18 (0.10 × 2.1, 2.6). Gradient elution; mobile phases: (A) water with 0.1% formic acid, (B) methanol with 0.1% formic acid. Ionization mode: positive/negative.	41 unifloral and 50 multifloral, from which 12 were honeydew honeys (Galicia, Spain)	For honeydew honey, gallic acid is the main chemical marker along with β-resorcylic acid and protocatechuic acid for their differentiation.	[75]
UAE followed by SPE	UHPLC–DAD-MS-MS. Syncronis C18 (0.100 × 2.1, 1.7). Gradient elution; mobile phases: (A) water with 0.1% formic acid, (B) acetonitrile. Ionization mode: negative.	4 unifloral, 5 honeydew, and 18 multifloral honeys (Tara, Serbia)	Nineteen phenolic compounds were characterized: six phenolic acids and thirteen flavonoids and their glycosides, the major polyphenols being p-coumaric acid, followed by caffeic acid and pinocembrin.	[76]
UAE followed by SPE	UHPLC–DAD-MS-MS. Syncronis C18 (0.100 × 2.1, 1.7). Gradient elution; mobile phases: (A) water with 0.1% formic acid, (B) acetonitrile. Ionization mode: negative.	20 blossom and 9 honeydew honeys (Montenegro, Serbia)	There were 32 characterized phenolic compounds: 14 flavonoids, 9 phenolic acids, and 9 derivatives. Luteolin and quercetin-3-O-galactoside were highlighted as differentiators of honeydew and polyfloral honeys.	[77]
SPE	LC-LRMS. Kinetex^®^ C18 porous-shell (0.1 × 4.6, 2.6) partially porous particle size). Gradient elution; mobile phases: (A) water with 0.1% formic acid, (B) acetonitrile. Ionization mode: negative.	34 multifloral, 76 blossom, 26 honeydew honeys (Spain)	There were 53 monitored phenolic compounds, from which only 35 were detected in honey samples. No specific polyphenols were associated with honeydew samples.	[78]
SPE followed by LLE	HPLC-MS. HALO C18 (0.50 × 4.6, 2.7). Gradient elution; mobile phases: (A) water with 0.1% formic acid, (B) acetonitrile with 0.1% formic acid. Ionization mode: positive/negative.	58 oak honeydew honeys (Spain)	Of 23 phenolic compounds identified, there were 7 benzoic acids, 4 hydroxycinnamic acids, and 12 flavonoids, with 16 being quantified and 6 being identified as biomarkers of geographical origin (salicylic acid, p-coumaric acid, p-hydroxybenzoic acid, syringic acid, naringenin, and galangin).	[79]
SPME	GC-MS. J&W DB-5HT (30 × 0.32, 0.1). Carrier gas: He.	2 chestnut (France), 2 fir (France, Italy), 2 acacia (France, Hungary), 2 Pyrenees (France), 2 orange (Spain, Italy), 2 lavender (France), 2 eucalyptus (Italy, Spain), 1 forest (Italy), 1 oak (France)	Thirty-one phenolic and volatile compounds were identified and quantified, with salicylic acid being a biomarker to distinguish honeydew from nectar honey samples.	[80]
SPE	UHPLC-LTQ OrbiTrapMS (identification). Syncronis C18 (0.10 × 2.1, 1.7). Gradient elution; mobile phases: (A) water with 0.1% acetic acid, (B) acetonitrile. Ionization mode: negative. UHPLC-DAD-MS-MS (quantification). Syncronis C18 (0.10 × 2.1, 1.7). Gradient elution; mobile phases: (A) water with 0.1% acetic acid, (B) acetonitrile Ionization mode: negative.	22 silver fir, 15 evergreen oak, 4 Hungarian oak, 6 Montpellier maple, 17 conifers honeydew honeys (Croatia)	Fifty-two phenolic compounds were identified through an SPE-UHPLC-LTQ OrbiTrapMS method, with twenty-five of these quantified through an SPE-UHPLC-DAD-MS-MS method. A pattern recognition analysis of the data from the phenolic compounds revealed that quercetin, naringenin, caffeoylquinic acid, hydroxyphenylacetic acid, apigenin, and genistein could be considered as potential markers of the botanical origin of honeydew honey.	[81]

^a^ Column model, length, m × internal diameter, mm; film thickness, µm.

**Table 4:** MS-based methods for the classification of honeydew honeys and blossom honeys by elemental fingerprinting.

Method(s)	Samples (Country of Origin)	Elements	Main Outcomes	Reference
ICP-MS FAAS	21 honeydew, 19 buckwheat, and 15 rape honeys (Poland)	Ca, Cu, Fe, K, Mg, Na, and Zn (FAAS); Al, B, Ba, Cd, Cr, Mn, and Ni (ICP-MS)	The clear discrimination of honeydew and nectar honeys was revealed by cluster analysis.	[30]
ICP-MS	34 Bracatinga honeydew honeys (Brazil)	Al, As, Au, Ba, Ce, Co, Cr, Cs, Cu, Dy, Eu, Er, Fe, Gd, Ho, In, Ir, La, Lu, Mg, Mn, Nd, Pb, Pd, Pr, Pt, Rb, Sb, Se, Sm, Tb, Te, Th, Tl, Tm, U, V, Yb, and Zn	The concentrations of main and trace elements were more effective than those of REEs in grouping the samples; indeed, Rb and Co were the main descriptors in PCA. The LDA correctly classified 91.3% of the samples of Bracatinga honeydew honey according to their geographic origin.	[89]
ICP-MS	7 acacia, 8 buckwheat, 10 coniferous honeydew, 1 fir, 7 heather, 8 linden, 9 nectar honeydew, 7 rape (Poland) 2 arbutus, 2 chestnut, 1 fir, 3 heather, 15 multifloral, 1 orange, 1 pine, 4 thyme (Greece)	Y, La, Ce, Pr, Nd, Sm, Eu, Gd, Tb, Dy, Ho, Er, Tm, Yb, Lu, Li, Mg, Mn, Ni, Co, Cu, Sr, Ba, and Pb	CA classified honeys according to their botanical origin, DA classified honeys according to botanical and geographical origins, and PNN and PLS classified honeys according to geographical origin. No algorithm was able to distinguish organic honey from conventional honey.	[90]
ICP-MS FAAS	37 honeydew, 39 buckwheat, and 58 rape honeys (Poland)	Ca, Cu, Fe, K, Mg, Na, and Zn (FAAS); Al, B, Ba, Cd, Cr, Mn, and Ni (ICP-MS)	LDA and C&RT allowed for an accurate attribution of the botanical origin of each honey. No algorithm contemporarily classified botanical and geographical origins. LDA classified the geographical origin inside each class of honey using Mg, Al, and Mn as descriptors.	[91]
ICP-MS FAAS	19 honeydew, 11 rape honeys (Poland)	Ca, Cu, Fe, K, Mg, Na, and Zn (FAAS); Al, B, Cr, Mn, and Ni (ICP-MS)	Cluster analysis revealed correlations between elemental concentrations and botanical origin. Honeydew honey exhibited higher concentrations of Mn, Al, Cu, K, Fe, and Ni than rape honey.	[94]
ICP-OES ICP-MS	22 silver fir, 15 evergreen oak, 4 Hungarian oak, 6 Montpellier maple, 17 conifers honeydew honeys (Croatia)	Al, Ca, Fe, K, Mg (ICP-OES), and As, Ba, Cd, Co, Cr, Cu, Hg, Mn, Ni, Pb, Se, Sr, and Zn (ICP-MS)	No contemporary classification of botanical and geographical origins has been achieved in this case. PCA revealed the differentiation of Hungarian oak honeydew honeys from the remaining samples using Mg, Mn, and Ba as descriptors, while no geographic classification was achieved.	[95]
ICP-MS	8 multifloral, 3 honeydew, 10 heather, 2 sage, 2 bearberry, 3 Mandarin orange honeys (Croatia)	Al, Ba, Be, Ca, Cd, Co, Cr, Cu, Fe, K, Mg, Mn, Mo, Na, Ni, Pb, Se, Sb, Th, U, V, and Zn	The honeydew honey samples exhibited the highest concentrations of Al, As, Be, Cd, Co, Cu, K, Mn, Ni, Sb, Th, U, V, and Zn.	[96]
ICP-MS EA-IRMS	18 acacia, 9 linden, 6 honeydew, 6 colza, 6 sunflower, 1 coriander, 1 yellow bedstraw, 1 thyme, 1 raspberry, 1 Amorpha honeys (Romania) 18 acacia, 3 linden, 6 honeydew, 6 colza, 5 sunflower, 6 lavender, 5 chestnut, 2 coriander (France)	Fifty-six, including main, trace, and toxic elements and REEs δ^2^H and δ^18^O of the water extracted from honey, δ^13^C from raw honey, δ^13^C and D/H ratios from the ethanol obtained through honey fermentation	The D/H ratio, K, V, Cr, As, Nb, δ^2^H, Ce, and δ^13^C are effective in the LDA model in classifying the geographical origin of honeys. No classifying errors were found within each botanical origin.	[97]
ICP-MS	9 acacia, 9 Tilia, 9 sunflower, 9 honeydew honeys (Romania)	Ag, Al, As, Ba, Be, Ca, Cd, Co, Cr, Cs, Cu, Fe, Ga, K, Li, Mg, Mn, Na, Ni, Pb, Rb, Se, Sr, Tl, U, V, and Zn	The chemometric approach used allowed the complete discrimination of the botanical origins of the samples.	[98]
ICP-AES IRMS	112 multifloral, 60 acacia, 37 chestnut, 18 citrus, 15 rhododendron, 13 eucalyptus, 10 honeydew honeys (Italy)	Al, B, Ba, Ca, Cr, Cu, Fe, K, Mg, Mn, Na, Ni, Pb, Rb, Sr, and Zn δ^13^C	IRMS measurements allowed the differentiation of honeys according to their botanical origin. No geographical differentiation was performed.	[99]
ICP-MS	Acacia, chestnut, country flowers, lime tree, multiflora, orange tree, rosemary, strawberry, and honeydew honeys (Italy)	Pb and Cd	Reproducibility was identified as the main source contributing to the overall uncertainty of the method. Honeydew honey samples present the highest amounts of Cd and Pb.	[100]

**Table 5:** Selected GC-MS-based studies for the investigation of the volatile profiles of honeydew honeys.

Extraction Technique	Chromatographic Conditions ^a^	Samples (Country of Origin)	Main Outcomes	Reference
Microscale SDE	BP-21, 50 × 0.32, 0.32	3 holm oak, 2 oak, 4 forest honeydew honeys (Spain)	Trans-oak lactone has been proposed as a potential marker for oak honeydews. Aminoacetophenone and propylanisole were identified as characteristic compounds for holm oak honeydews.	[107]
USE and HS-SPME	HP-5MS, 30 × 0.25, 0.25	2 oak (*Quercus frainetto* Ten.) honeydew honeys (Croatia)	HS-SPME enabled the identification of the most volatile compounds, which were dominated by terpenes such as cis- and trans-linalool oxides. USE can also detect less-volatile organic compounds, such as phenylacetic acid. These three compounds are the most representative of the volatile fraction of oak honeydew honey.	[122]
USE and HS-SPME	HP-5MS, 30 × 0.25, 0.25 HP-FFAP, 50 × 0.32, 0.50	1 *Salix* spp. honeydew honey (Croatia)	The volatile composition of Salix honeydew honey from Croatia is distinguished by the presence of high amounts of benzoic acid, phenylacetic acid, 2-hydroxybenzoic acid, and 4-hydroxyphenylacetic acid.	[123]
USE and HS-SPME	HP-5MS, 30 × 0.25, 0.25	5 fir honeydew honeys (Poland)	The volatile fraction of fir honeydew honey from Poland is primarily constituted by higher aliphatic hydrocarbons and alcohols, phenylpropanoids (UAE), and benzaldehyde, phenylacetaldehyde, and linalool derivatives (SPME). The main volatile species found in fir honeydew honeys from other countries are not abundant in this study. Protocatechuic acid has been proposed as a marker for fir honeydew honeys.	[125]
HS	Ion flow tube–mass spectrometry. No chromatographic separation was used in this case.	8 unifloral blossom honeys and 1 beech honeydew honey (New Zealand)	The method enables the differentiation between manuka honey and its most prevalent contaminant, namely, beech honeydew honey.	[105]
HS	Proton-transfer-reaction time-of-flight-mass spectrometry. No chromatographic separation was used in this case.	70 honeys of citrus, chestnut, sunflower, honeydew, robinia, rhododendron, and linden tree (Italy)	The LDA-PNN treatment of the data obtained permitted the correct classification of honeys according to their botanical origin.	[106]
Purge and trap	SGE BPX5, 30 × 0.25, 0.25	44 pine honeydew honey (22 Greece, 22 Turkey)	3-Carene was present in all samples from Turkey but absent in all samples from Greece, whereas the exogenous species 1,4-dichlorobenzene was found exclusively in all samples from Greece and not in any samples from Turkey.	[108]
SPME	19081S-433 HP5MS, 30 × 0.25, 0.25	24 pine honeydew honeys (Turkey)	16-Oxosalutaridine was proposed as a potential marker of authenticity for this honey. 3-Carene, proposed as a marker of geographical origin in [50], was never found in this study.	[109]
HS-SPME	DB-5MS, 60 × 0.32, 1	34 *Quercus ilex* honeydew honeys (Greece)	Eucalyptol, 1-decanol, and tetradecanoic acid ethyl ester are proposed as volatile indicators of the provenance of Greek *Quercus ilex* honey. The geographical origin was determined through melissopalynological analysis.	[110]
HS-SPME	DB-5MS, 60 × 0.32, 1	1 honeydew honey (Greece)	Significant variation in the composition of 2-butanone, 2-methylpropanal, ethyl acetate, and α-pinene was observed over the course of one year under in-house conditions.	[111]
HS-SPME	HP-INNOWax, 30 × 0.32, 0.5	3 honeydew, 5 lime tree, and 9 sage honeys (Croatia)	Differentiation among Croatia fir honeydew honey and sage and lime blossom honeys was reported. Honeydew honey contains acetonitrile, methyl-2-buten-1-ol, n-hexanol, 3-hexanol, 1-propyne, 2-furanmethanol, 5-methyl-2(5H)-furanone, 4-methylphenol, hexadecanoic acid, and methylheptanoate, which are not found in blossom honeys.	[112]
SPME (1) PA fiber (2) C/PDMS fiber	(1) A Carbowax 20 M, 50 × 0.25, 0.25 (2) HP-Innowax, 50 × 0.20, 0.20	1 fir honeydew, 1 *Quercus ilex* honeydew, 11 blossom honeys (Spain)	A discrimination analysis was conducted on Spanish honeydew honey samples and eleven blossom honeys based on the presence of α-pinene, α-phellandrene, and eucalyptol.	[55]
Purge and trap	Supelcowax-10, 50 × 0.25, 0.25	4 honeydew honeys, 6 multifloral honeys and 12 unifloral honeys (Spain)	The differentiation between Croatian fir honeydew honey and sage and lime blossom honeys was successfully accomplished. Honeydew honey contains acetonitrile, methyl-2-buten-1-ol, n-hexanol, 3-hexanol, 1-propyne, 2-furanmethanol, 5-methyl-2(5H)-furanone, 4-methylphenol, hexadecanoic acid, and methylheptanoate. None of these species have been found in blossom honeys.	[113]
HS-SPME	HP-5MS, 30 × 0.25, 0.25	64 blossom honeys, 6 honeydew honeys (Turkey)	Nonanal and, in particular, α,α-dimethylphenylacetate appear to be promising markers of the botanical origin of honeydew honey from Turkey. α,α-Dimethylphenylacetate was not identified in previous studies, and in this study, other potential markers of the botanical origin of Turkish honeys, such as 3-carene or 16-oxosalutaridine, were not found.	[60]
HS-SPME	GCxGC Primary phase: VF1-MS, 30 × 0.25, 1 Secondary phase: SolGel-Wax, 1.5 × 0.25, 0.25	5 unifloral blossom and 1 honeydew honey (Poland)	(E)-Nonen-2-al has been proposed as a potential marker for identifying the botanical origin of Polish honeydew honey. A combination of four ethyl esters, 2,3-dimethylphenol, and 2,3-dimethylpyrazine allowed for the classification of the five samples based on their geographical origin.	[114]
HS-SPME	GCxGC Primary phase: DB-5ms, 30 × 0.25, 0.25 Secondary phase: Supelcowax-10, 1.2 × 0.1, 0.1	35 honeydew honeys (Slovakia)	The presence of 2-oxooctanoic acid, 4-oxapentanoic acid, allyl ester of acetic acid, and methyl ester of 2,6-dihydroxybenzoic acid, which are always present in honeydew honey and never identified in blossom honeys, has been suggested as a potential marker of origin for Slovakian honeydew honey.	[115]
HS-SPME	DB-5MS, 60 × 0.32, 1	49 blossom honeys, 31 fir honeydew honey, 39 pine honeydew honey (Greece)	The botanical discrimination of two honeydew honeys and three unifloral blossom honeys was accomplished according to different amounts of C6-C14 ethyl esters. The exogenous species 1,4-dichlorobenzene, proposed by Takanaki [49] as a potential marker of Greek honeys, was not identified in this sampling.	[116]
HS-SPME	HP5MS, 30 × 0.25, 1	1 dandelion, 1 fir tree, 1 linden tree, 1 chestnut tree, 1 robinia, 1 orange, 1 lavender, and 1 rape honeys (Austria and Croatia)	PCA unambiguously differentiates the cluster comprising the three honeydew honeys (fir, lime, and chestnut) from that formed by the blossom honeys. The concentration of butanoic acid, diacetyl, and 2-aminoacetophenone is correlated with the honeydew honeys.	[117]
HS-SPME	Rtx-1 (PDMS), 30 × 0.25, 1	269 honey samples, including 48 honeydew maquis (Corsica, France)	The concentration of 3-furaldehyde was found to be a discriminant between blossom and honeydew honeys. This is the first report of the volatile profile of *Metcalfa* honeydew honey.	[118]
HS-SPME	DB-5MS, 60 × 0.32, 1	26 blossom honey and 6 honeydew honey (Greece)	Low amounts of C8-C10 linear aldehydes, ethyl esters, and alkanes, furfural, thymol, and α-pinene have been identified, yet none of these species are exclusive to this honey.	[119]
HS-SPME	Direct Injection Mass Spectrometry. No chromatographic separation was used in this case.	4 acacia, 4 citrus, 5 eucalyptus, 5 honeydew and 4 rosemary honeys (Spain)	PLS-LDA was employed to classify the samples according to their botanical origin. The specific volatile fingerprint of honeydew honey is characterized by high amounts of 2-phenylethanol, isophorone, 2-methyl-1-butanol, and 3-methyl-1-butanol. Dimethylacetophenone is a constituent of honeydew honeys that is absent in blossom honeys.	[120]
SPME Arrow	SUPELCOWAX™ 10, 30 × 0.25, 0.25 and SLB-5 (2.0 × 0.10, 0.10)	1 nectar, 1 honeydew, 1 pine honeys (Greece and Austria)	High concentrations of nonanal and nonanoic acid were observed in pine honeys. Forest honeydews exhibited high concentrations of octanoic acid, 3,5,5-trimethylcyclohex-2-enone, and α-methyl-α-[4-methyl-3-pentenyl] oxiranemethanol.	[121]
USE and HS-SPME	HP-5MS, 30 × 0.25, 0.25	1 fir honeydew honey (Croatia)	The volatile fraction of fir honeydew honey is primarily constituted by phenylacetic acid, 4-hydroxyphenylacetic acid, 4-hydroxycinnamic acid, hexadecane-1-ol, and palmitic acid.	[124]

^a^ Column model, length, m × internal diameter, mm; film thickness, µm.

**Table 6:** Selected MS-based studies on the determination of contaminants and toxins in honeydew honeys.

Method(s)	Chromatographic Conditions ^a^	Analytes	Samples (Country of Origin)	Main Outcomes	Reference
GC-MS	The QuEChERS method was used for the extraction of the pesticides. GC column: Agilent DB-5MS (30 m length × 0.25 mm internal diameter × 0.2 µm particle size).	Atrazine, chlorpyrifos, α-endosulfan, τ-fluvalinate, chlorfenvinphos, chlorfenvinphos, bromopropylate, and coumaphos	28 Bracatinga honeydew honeys (Brazil)	Traces of τ-fluvalinate were found in only one honey sample, while low levels of atrazine were found in some samples produced in the state of Santa Catarina. In both cases, the amounts were less than the limits imposed for honeys.	[43]
LC-MS-MS	LC column: Xbridge C18 (100 mm length × 2.1 mm internal diameter, 3.5 μm particle size). Gradient elution; mobile phases: (A) water + 0.1% formic acid, (B) acetonitrile + 0.1% formic acid. Ionization mode: positive.	Imidacloprid and spirotetramat	Soil, foliage, and honeydew honeys (USA)	Both insecticides reduce the amount of honeydew produced and are present in their unmetabolized forms in soils and in foliage. It is possible that both insecticides bioaccumulate in honeydew and potentially harm honeydew-feeding organisms, like bees.	[126]
LC-MS-MS	LC column: Luna C18 (150 mm length × 2.1 mm internal diameter, 3 μm particle size). Isocratic elution (80% A, 20%B); mobile phases: (A) water + 0.1% formic acid, (B) methanol + 0.1% formic acid. Ionization mode: positive.	Pymetrozine and flonicamid	Honeydew honeys (Spain)	The presence of insecticides in hemipteran honeydew depends on their species. Honeydew-producing species resistant to insecticides may excrete contaminated honeydew for longer periods of time. Hence, the environmental risk to beneficial insects and consumers of honeydew-based products is higher than that posed by contaminated nectar.	[127]
LC-MS-MS	The QuEChERS method was used for the extraction of the analytes. LC column: Poroshell 120 EC-C18 (50 mm length × 4.6 mm internal diameter, 2.7 μm particle size). Isocratic elution (85% A, 15% B). Mobile phases: (A) methanol; (B) water with 0.1% formic acid.	Dinotefuran	Three samples each of worker bees, wax, and honeydew honey and samples of lanternfly honeydew (New Zealand)	None of the worker bee, wax, or honey samples indicated detectable levels of dinotefuran; however, honeydew samples collected did contain dinotefuran above the detection limit, with amounts ranging from 3 to 100 ng per sample.	[128]
LC-MS-MS NMR	LC column: Poroshell 120 SR-C18 (150 mm length × 2.1 mm internal diameter, 2.7 μm particle size). Gradient elution; mobile phases: (A) 10 mM aqueous NH_4_COOH, (B) methanol. Ionization mode: negative. NMR was used to characterize the tutin-derived structures.	Tutin and its glycoside derivatives	Three samples of honeydew honey and *Coriaria* leaf samples (New Zealand)	Tutin and its derivatives were quantified at levels of mg/kg.	[129]
LC-MS-MS	LC column: Luna 3μ-C18 (100 mm length × 2 mm internal diameter, 3 μm particle size). Gradient elution; mobile phases: (A) 10 mM aqueous NH_4_COOH, (B) methanol. Ionization mode: negative.	Tutin, hyenanchin, and relevant glycoside derivatives	Three honeydew honeys, honeydews, different parts of the Tutu shrub, and *Scolypopa australis* hemitters (New Zealand)	The study confirmed that all of these picrotoxanes, including tutin derivatives, are of plant origin and not metabolites produced by the insect.	[130]

^a^ Column model, length, m × internal diameter, mm; film thickness, µm.

## Data Availability

No new data were created or analyzed in this study. Data sharing is not applicable to this article.
